# The strength of Nesterov’s accelerated gradient in boosting transferability of stealthy adversarial attacks

**DOI:** 10.1371/journal.pone.0337463

**Published:** 2025-11-25

**Authors:** Chen Lin, Sheng Long

**Affiliations:** 1 Joint Laboratory of Data Science and Business Intelligence, Southwestern University of Finance and Economics, Chengdu, Sichuan, China; 2 Laboratory for Big Data and Decision, National University of Defense Technology, Changsha, Hunan, China; Najran University College of Computer Science and Information Systems, SAUDI ARABIA

## Abstract

Deep neural networks have been shown to be highly vulnerable to adversarial examples—inputs crafted to mislead models by adding subtle, human-imperceptible perturbations. Transferability and stealthiness are two crucial metrics for evaluating adversarial attacks. However, these goals often conflict: examples with high transferability typically exhibit noticeable adversarial noise, while those with imperceptible perturbations tend to perform poorly in black-box attacks. To tackle this, we propose Diff-AdaNAG, a novel framework that introduces Nesterov’s Accelerated Gradient (NAG) into diffusion-based adversarial example generation. Specifically, the diffusion mechanism guides the generation process toward the natural data distribution, achieving stealthy attacks with imperceptible adversarial examples. Meanwhile, an adaptive step-size strategy is utilized to harness the strong acceleration and generalization capabilities of NAG in optimization, enhancing black-box transferability in adversarial attacks. Extensive experiments demonstrate that Diff-AdaNAG consistently outperforms state-of-the-art methods in both white-box and black-box scenarios, significantly boosting transferability without compromising stealthiness. The code is available at https://github.com/Linc2021/Diff-AdaNAG.

## Introduction

Deep neural networks are vulnerable to adversarial attacks—subtle perturbations that are visually indistinguishable from natural data yet capable of misleading model predictions [1,2]. This vulnerability poses serious security risks in real-world applications, making robustness against such attacks a critical research problem. Understanding the mechanisms behind adversarial attacks is essential for developing effective defenses. Typically, adversarial example generation is formulated as a constrained optimization problem, including sign-based methods such as Fast Gradient Sign Method (FGSM) [1], Basic Iterative Method (BIM) [3], Projected Gradient Descent (PGD) [4], etc. While PGD performs well in white-box scenarios, its performance declines in black-box contexts, often underperforming compared to FGSM. Consequently, various PGD-inspired algorithms have been proposed, highlighting the importance of rigorously evaluating these methods to enhance model robustness and security.

Among evaluation metrics, transferability and stealthiness stand out as the two core criteria for assessing the effectiveness of adversarial attacks. Transferability refers to the ability of adversarial examples to generalize across different models, enabling successful attacks even when the attacker has incomplete knowledge of the target model’s structure and parameters. Stealthiness, on the other hand, demands that adversarial examples be visually indistinguishable from original images to avoid human detection and being easily identified and filtered by defense mechanisms. In practice, however, there is often a trade-off between these two metrics. Algorithms that focus on generating imperceptible adversarial examples often exhibit poor performance in black-box attack scenarios, with their transferability significantly limited [5]. In contrast, some algorithms enhance transferability by introducing more noticeable adversarial noise at the expense of stealthiness [6].

To further improve the stealthiness of adversarial attacks, diffusion-based mechanisms have been introduced to generate adversarial examples. DiffAttack employs the Denoising Diffusion Implicit Model (DDIM) inversion to project clean images into the diffusion latent space and add noise there; this latent space alteration yields adversarial examples that are visually nearly indistinguishable from the original images [7]. Recently, Diff-PGD [8] exploits a diffusion mechanism to generate adversarial examples that are closer to the natural data distribution, improving the stealthiness and effectiveness of the attack. Similar work includes the NS-Diff-PGD presented by [9]. However, Diff-PGD focuses on producing visually stealthy adversarial examples and suffers from a low attack success rate in black-box settings.

Addressing these limitations, numerous effective strategies exist for training adversarial examples with transferability on a white-box surrogate model. In particular, representative algorithms derived from FGSM, namely MI-FGSM [10] and NI-FGSM [11], correspond to Heavy-Ball (HB) [12] and Nesterov’s Accelerated Gradient (NAG) [13], respectively. Notably, in traditional optimization fields, NAG is a more advanced momentum algorithm than HB. NI-FGSM, which incorporates the NAG mechanism, demonstrates remarkable black-box transferability. However, MI-FGSM and NI-FGSM still rely on the sign-based optimization used in FGSM and PGD. The use of the sign function may result in uncontrollable algorithmic non-convergence [14] and poor generalization [15]. Therefore, we tend to believe that the potential of NAG hasn’t been fully exploited. To address this concern, an adaptive step-size strategy is used to improve MI-FGSM [16]. It evades the adverse effects of the sign function and generates adversarial noise that is both transferable and imperceptible.

Motivated by these insights, we propose Diff-AdaNAG, an innovative adversarial attack framework that synergistically integrates Adaptive Nesterov’s Accelerated Gradient optimizer (AdaNAG) with diffusion-based adversarial example generation inspired by Diff-PGD. By replacing problematic sign-based operations with adaptive NAG momentum, Diff-AdaNAG establishes the direct connection of NAG in convex optimization theory and adversarial attack practice, unleashing its strength in black-box transferable attacks. Additionally, the diffusion mechanism significantly increases the stealthiness and effectiveness of black-box adversarial attacks by generating examples closer to the natural data distribution. Comprehensive experiments demonstrate that Diff-AdaNAG consistently outperforms state-of-the-art methods under both white-box and black-box scenarios, highlighting its superior attack success rates and improved transferability. Our contributions are as follows.

- We propose an innovative adversarial attack algorithm that produces stealthy adversarial examples by a diffusion-based mechanism inspired by Diff-PGD.
- We introduce adaptive NAG momentum to enhance controllability by replacing problematic sign-based operations with adaptive step-size.
- We conduct comprehensive experiments to show that the proposed method consistently outperforms state-of-the-art methods under both white-box and black-box scenarios, highlighting its superior attack success rates and improved transferability.

**Notation.** We use lower case bold letters **x** to denote vectors and upper case bold letters **X** to denote matrices. We use $\|\;\cdot \;{\|}_{p}$ to denote the $\ell_{p}$-norm of a vector, and $\|\;\cdot \;{\|}_{\infty }$ to denote the ℓ_∞_-norm. For any vectors a, $b\in {\mathbb{R}}^{d}$, all standard operations such as $ab,a^{2},a^{1/2}$ and 1/a are assumed to be element-wise. We use $\nabla_{𝐱}f(𝐱)$ to denote the gradient of a function *f* with respect to **x**. We use **I** to denote the identity matrix, and **0** to denote the zero vector.

## Related works

We aim to generate a non-targeted adversarial example ${𝐱}^{adv}$ from a given input ${𝐱}^{0}$ with the true label *y*. The goal is to minimize the objective function −L(𝐱,y), in order to maximize the discrepancy between the predicted label of the adversarial example and the true label. The optimization problem can be formulated as follows:

$${𝐱}^{adv}=\arg\min_{𝐱}-L(𝐱,y)\;\text{s.t.}\;\|𝐱-{𝐱}^{0}{\|}_{\infty }\leq \epsilon ,$$ (1)

where *ε* is the $\ell_{\infty }$-norm constraint on the perturbation. The loss function L(𝐱,y) is typically the cross-entropy loss between the predicted label of the adversarial example and the true label. We can learn adversarial examples using the gradient ascent direction in practice.

### Adversarial attacks

There are many methods to solve problem (1), such as FGSM [1], BIM [17], and PGD [4] (sign-based). FGSM generates adversarial examples by taking a single step in the direction of the sign of the gradient of the loss function with respect to the input:


$${𝐱}^{adv}={𝐱}^{0}+\epsilon \cdot \text{sign}(\nabla_{{𝐱}^{0}}L({𝐱}^{0},y)).$$ (**FGSM**)

BIM iteratively takes small steps in the direction of the gradient and projects the perturbed input back to a valid $\ell_{\infty }$-ball centered around the original input. Then, PGD is proposed as a multi-step variant of BIM by introducing random noise initialization.


$${𝐱}^{t+1}=\Pi_{{𝐱}^{0}+\epsilon }({𝐱}^{t}+\alpha_{t}\cdot \text{sign}(\nabla_{{𝐱}^{t}}L({𝐱}^{t},y))),$$ (**PGD**)

where $\Pi_{𝐱+\epsilon }({𝐱}^{t})$ projects ${𝐱}^{t}$ back to the $\ell_{\infty }$-ball centered around **x** with radius *ε* and $\alpha_{t}=\frac{\epsilon }{T}$. However, their attack effectiveness falls short in black-box scenarios. Dong et al. propose MI-FGSM to address this issue by incorporating momentum into the optimization process [10], then Lin et al. modify the gradient calculation position of MI-FGSM and proposed NI-FGSM to further improve the transferability of adversarial examples [11]:


$${𝐱}^{t+1}=\Pi_{{𝐱}^{0}+\epsilon }({𝐱}^{t}+\alpha_{t}\cdot \text{sign}(m^{t+1})),\;m^{t+1}=\mu m^{t}+\frac{\nabla_{{𝐱}^{t}}L({𝐱}^{t},y)}{||\nabla_{{𝐱}^{t}}L({𝐱}^{t},y)|{|}_{1}},$$ (**MI-FGSM**)

$$m^{t+1}=\mu m^{t}+\frac{\nabla_{{𝐱}^{t}}L({𝐱}^{t}+\alpha_{t}\mu \cdot m^{t},y)}{||\nabla_{{𝐱}^{t}}L({𝐱}^{t}+\alpha_{t}\mu \cdot m^{t},y)|{|}_{1}},$$ (**NI-FGSM**)

where ${𝐦}^{t}$ is the momentum term and *μ* is the momentum factor suggested as a constant value.

Nevertheless, these approaches still share a common drawback: they all incorporate the sign function into the iterative update process of adversarial samples. Multiple studies have demonstrated that reliance on the sign function may adversely affect the algorithm’s practical efficacy [14] and, in extreme cases, compromise its convergence guarantees [15]. Moreover, while NI-FGSM introduces extrapolation-based momentum updates into its iterative framework, this implementation does not maintain strict mathematical equivalence to the NAG method (which will be elaborated in the next section). This divergence suggests that NI-FGSM fails to harness the optimization advantages inherent to NAG. Consequently, its untapped potential in black-box transferable adversarial attacks warrants further exploration through refined algorithmic adaptations.

In addition to the generic gradient-based attack methods mentioned above, there are also some advanced feature-level techniques such as FIA [18], NEAA [19], and MFAA [20]. The core principle of these methods is to enhance transferability by selectively disrupting category-related and cross-model invariant features encoded in the intermediate layers of deep networks. By focusing on object-aware representations that consistently drive decisions across models, the resulting adversarial examples exhibit stronger transferability. In contrast, CAAM [21] improves transferability by exploiting cross-model channel redundancy and invariance, through channel shuffling/reweighting and channel-invariant blocks. Yet the same layer- or channel-level tampering that boosts transferability also leaves larger, more structured perturbation footprints, so stealth drops in exchange for higher black-box success.

### Diffusion models

Diffusion Models [22–24] show a significant performance in many fields, such as image generation [23], text-to-image generating [25–27], video generation [28–30], 3-D generation [31,32], and adversarial attacks [33–35].

The Denoised Diffusion Probabilistic Model (DDPM) [23] is a discretized variant of diffusion models. Let $x_{0}\sim p(x_{0})$ denote a sample from the natural image distribution. Through a forward diffusion process, Gaussian noise is incrementally added to **x**_0_, resulting in progressively noisier samples $\left[x_{1},x_{2},\ldots ,x_{t},\ldots ,x_{T}\right]$ over *T* steps, governed by the Markovian process:


$$q(x_{t}|x_{t-1})=𝒩(x_{t};\sqrt{1-\beta_{t}}x_{t-1},\beta_{t}𝐈),$$

where 𝒩 represents a Gaussian distribution, and $\beta_{t}$ increases from 0 to 1. The conditional probability $q(x_{t}|x_{0})$ is:


$$q(x_{t}|x_{0})=N(x_{t};\sqrt{{\bar{\alpha }}_{t}}x_{0},(1-{\bar{\alpha }}_{t})I),$$

where $\alpha_{t}=1-\beta_{t}$ and ${\bar{\alpha }}_{t}=\prod_{s=1}^{t}\alpha_{s}$. As ${\bar{\alpha }}_{t}$ approaches zero, $x_{T}$ converges to an isotropic Gaussian distribution.

The joint distribution $p_{\phi }({𝐱}_{0:T})$ is called the reverse process modeled by a Markov chain with learned Gaussian transitions, starting from $p({𝐱}_{T})=𝒩({𝐱}_{T};0,𝐈)$. It aims to reconstruct samples by refining Gaussian noise. The transition model, $p_{\phi }(x_{t-1}|x_{t})$, is optimized by minimizing the variational bound on the negative log-likelihood. A modified U-Net [23] is typically used for denoising. The reverse diffusion process is:


$$p_{\phi }({𝐱}_{0:T}):=p({𝐱}_{T})\prod_{t=1}^{T}p_{\phi }({𝐱}_{t-1}|{𝐱}_{t}),\;p_{\phi }({𝐱}_{t-1}|{𝐱}_{t}):=𝒩({𝐱}_{t-1};\mu_{\phi }\left({𝐱}_{t},t\right),\Sigma_{\phi }\left({𝐱}_{t},t\right)).$$

We can use $R_{\phi }$ to represent such a sampling process with $x_{t-1}=R_{\phi }(x_{t},t)$.

Diffusion models [23,36] are also used in guided image synthesis. Based on a diffusion model generative prior, SDEdit (Stochastic Differential Editing) [37] synthesizes realistic images by iteratively denoising via a stochastic differential equation. The key idea is to perturb the input sample according to $x_{K}\sim q(x_{K}|x)$ and then iteratively apply the learned denoising function $R_{\phi }$:


$$\text{SDEdit}(x,K)=R_{\phi }(\ldots R_{\phi }(R_{\phi }(x_{K},K),K-1)\ldots ,0).$$

SDEdit applies *K* forward diffusion steps, followed by *K* reverse steps to align the sample with the natural data distribution $p(x_{0})$. In adversarial learning, SDEdit enhances stealthiness, generating images that reside in a transitional space between the input and realistic data distributions. Several diffusion-based methods have been proposed for adversarial attacks [8,9,34]. These approaches typically involve applying SDEdit to image samples at each step of the gradient ascent procedure. In addition, such a mechanism can also be used in adversarial defense tasks [38,39].

## Methodology

**Fig 1:**
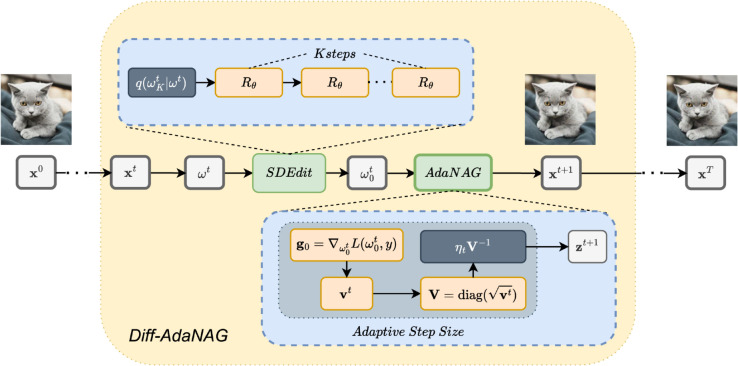
Our proposed Diff-AdaNAG framework. *q* is forward diffusion and $R_{\phi }$ is backward denoising, $\omega_{t}^{0}$ is the denoised sample.

In order to further strengthen the transferability of stealthy adversarial attacks, we present the Diff-AdaNAG algorithm for generating adversarial examples. The algorithm integrates the AdaNAG optimizer with a diffusion-based mechanism inspired by Diff-PGD. In the first subsection, we highlighted the differences between NAG and NI-FGSM. Then, we introduced AdaNAG to address this gap in the second subsection. Subsequently, in the third subsection, we proposed a diffusion-based AdaNAG method for achieving stealthy adversarial attacks. Finally, we presented an efficient accelerated algorithm for practical implementation in the last subsection. Fig 1 provides a high-level overview of the proposed Diff-AdaNAG algorithm.

### The gap between Nesterov’s accelerated gradient and NI-FGSM

NAG [13,40] is a popular momentum method that has been shown to converge faster than traditional gradient descent in smooth convex optimization, and its form is as follows:


$$\omega^{t}=(1-\theta_{t})x^{t}+\theta_{t}z^{t},\;z^{t+1}=\Pi_{{𝐱}^{0}+\epsilon }(z^{t}-L^{-1}\theta_{t}^{-1}\nabla_{\omega^{t}}L(\omega^{t},y)),\;x^{t+1}=(1-\theta_{t})x^{t}+\theta_{t}z^{t+1},$$ (**NAG**)

where $z^{0}=x^{0}$, *L* is the Lipschitz constant satisfying *L* > 0, $\theta_{t}$ is the momentum parameter satisfying $\frac{1-\theta_{k+1}}{\theta_{k+1}^{2}}\leq \frac{1}{\theta_{k}^{2}}$. From the second line of the algorithm, we can observe that the NAG method distinguishes itself by strategically shifting the gradient computation to the extrapolated point $\omega^{t}$ rather than the current position $x^{t}$. This adjustment is pivotal as it allows the algorithm to effectively harness the momentum from previous iterations, thereby accelerating convergence.

NI-FGSM adopts a similar concept but introduces an additional *L*_1_-norm normalization step for calculating the momentum term,


$$m^{t+1}=\mu m^{t}+\frac{\nabla_{{𝐱}^{t}}L({𝐱}^{t}+\alpha_{t}\mu \cdot m^{t},y)}{||\nabla_{{𝐱}^{t}}L({𝐱}^{t}+\alpha_{t}\mu \cdot m^{t},y)|{|}_{1}},$$

and employs the sign function to adjust the algorithm’s final update direction,


$${𝐱}^{t+1}=\Pi_{{𝐱}^{0}+\epsilon }({𝐱}^{t}+\alpha_{t}\cdot \text{sign}(m^{t+1})).$$

However, these operations, causing the lack of strict mathematical equivalence between NI-FGSM and NAG carry the risk of distorting gradient information, which may lead to suboptimal algorithmic performance. This further exacerbates the gap between theoretical convergence in optimization (NAG) and practical effectiveness in adversarial attacks (NI-FGSM).

### Adaptive Nesterov’s accelerated gradient

Fortunately, sign-based methods are closely connected to Adam-type methods [16]. Therefore, adaptive step-sizes offer a viable alternative to mitigate the potential drawbacks associated with the sign function. By incorporating it into the iterative steps of NAG, we propose the AdaNAG method:


$$\omega^{t}=(1-\theta_{t})x^{t}+\theta_{t}z^{t},\;v^{t}=v^{t-1}+(g⊙g)+\delta ,\;V=\text{diag}(\sqrt{v^{t}}),\;z^{t+1}=\Pi_{{𝐱}^{0}+\epsilon }(z^{t}-\eta_{t}\theta_{t}^{-1}V^{-1}g),\;x^{t+1}=(1-\theta_{t})x^{t}+\theta_{t}z^{t+1},$$ (**AdaNAG**)

where $g=\nabla_{\omega^{t}}L(\omega^{t},y)$, $z^{0}=x^{0}$, $\eta_{t}=\eta /(t+2)$ with η>0 and *δ* is a small constant to avoid division by zero. As we can see, unlike the step size strategy *L*^−1^ used in NAG for smooth optimization problems or the sign function employed in NI-FGSM for adversarial attacks, AdaNAG introduces an adaptive strategy $\eta_{t}V^{-1}$, which accumulates historical squared gradient information. Here, $V^{-1}$ is a diagonal matrix, with its diagonal entries representing the updated weights for the parameters in each dimension. Compared to the sign function, these entries may preserve more useful information. Moreover, given a clean image **x** and its corresponding label *y*, this method can also be used to generate adversarial examples. Consequently, AdaNAG bridges the gap between convex optimization theory and adversarial attack practice and, on the other hand, enhances interpretability by replacing problematic sign-based operations with adaptive step-sizes.

### Diffusion-based adaptive Nesterov’s accelerated gradient

To further enhance stealthiness, we integrate a diffusion mechanism into AdaNAG. In the update process of AdaNAG, we first obtain the weighted form of the perturbed image $\omega^{t}$ by linearly interpolating between the perturbed image $z^{t}$ and the clean image $x^{t}$ with a weight $\theta_{t}$ by $\omega^{t}=(1\;-\;\theta_{t})x^{t}\;+\;\theta_{t}z^{t}$. Then we calculate the gradient of the loss function $L(\omega^{t},y)$ with respect to the perturbed image $\omega^{t}$ and update $z^{t+1}$ along the gradient ascent direction:


$$z^{t+1}=\Pi_{{𝐱}^{0}+\epsilon }(z^{t}+\eta_{t}\theta_{t}^{-1}V^{-1}\nabla_{\omega^{t}}L(\omega^{t},y)).$$

In the spirit of Diff-PGD, we turn to use the purified image $\omega_{0}^{t}$ obtained by using SDEdit with *K* reverse steps. Then the update step of $z^{t}$ in Diff-AdaNAG become


$$\omega_{0}^{t}=\text{SDEdit}(\omega^{t},K),\;z^{t+1}=\Pi_{{𝐱}^{0}+\epsilon }(z^{t}+\eta_{t}\theta_{t}^{-1}V^{-1}\nabla_{\omega^{t}}L(\omega_{0}^{t},y)).$$ (**Diff-AdaNAG**)

It can be observed that when computing the gradient, differentiation is taken with respect to *y*^(*t*)^. This means we need to compute the adversarial gradient


$$g=\nabla_{\omega^{t}}L(\omega_{0}^{t},y)=\nabla_{\omega^{t}}L(R_{\phi }(\ldots R_{\phi }(R_{\phi }(\omega^{t},K),K-1)\ldots ,0),y)$$

through back-propagation. For such a reason, *K* cannot be too large due to GPU memory constraints.

### Accelerated diff-AdaNAG with gradient approximation

To improve computational efficiency, we adopt the memory-saving gradient approximation strategy proposed in [32,41,42] along with fast sampling techniques [36]. Instead of backpropagating through the full *K*-step SDEdit process, which incurs high GPU memory costs, we approximate the Jacobian of *K*-step outputs with a constant *c*, leading to the simplification $\partial \omega_{0}^{t}/\partial \omega^{t}\approx c$. This allows us to compute the gradient only at $\omega_{0}^{t}$ without storing intermediate gradients


$$\frac{\partial L(f(\omega_{0}^{t}))}{\partial \omega^{t}}\approx c\frac{\partial L(f(\omega_{0}^{t}))}{\partial \omega_{0}^{t}}.$$

So the adversarial gradient can be computed by


$$g_{0}=\nabla_{\omega_{0}^{t}}L(\omega_{0}^{t},y).$$

Additionally, we accelerate inference by leveraging the DDIM [36] sampling strategy, which sub-samples the original diffusion steps (*T*) into a reduced set (*T*_*s*_). Instead of using the full *K* steps in SDEdit, we scale it down to $K_{s}\ll K$, significantly improving efficiency (e.g., $T=1000,K=100,T_{s}=50,K_{s}=1$). This reduces the number of function evaluations while preserving the effectiveness of SDEdit.

By leveraging gradient approximation alongside DDIM acceleration, our method achieves substantial savings in both computation time and VRAM usage since it operates without gradient computation and solely relies on the DDIM inference model, all while maintaining a high success rate in global attack tasks. Finally, the algorithm 1 shows our method in its entirety.

**Algorithm 1 Diff-AdaNAG.**



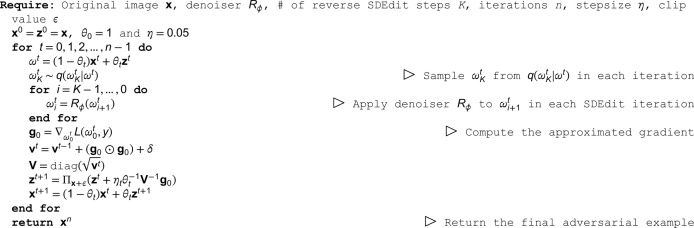



## Experiments

### Experimental setting

**Datasets.** We evaluate our Diff-AdaNAG algorithm on the validation dataset of ImageNet [43], which randomly selects 500 images from the ILSVRC 2012 validation set (https://www.image-net.org/challenges/LSVRC/2012/index.php).

**Models.** In order to demonstrate the superiority of the method, we conduct experiments on different models as much as possible. These models include: a series of ResNet models [44] including ResNet-18 (Res-18), ResNet-34 (Res-34), ResNet-50 (Res-50), and ResNet-101 (Res-101), EfficientNet-b0 (EffNet) [45], GoogLeNet (GooLeNet) [46], Inception-v3 (Inc-v3) [47], MNASNet0-5 (MNAS) [48], MobileNet-v3-small (MobNet) [49], ShuffleNet-v2-x0-5 (ShufNet) [50], SqueezeNet-1-1 (SqueNet) [51] and VGG11 (VGG-11) [52]. These models are all pre-trained models from the torchvision library [53] and are used as both source models for generating adversarial examples and target models for testing these adversarial examples.

**Baselines.** We compare the proposed Diff-AdaNAG algorithm with several state-of-the-art adversarial attacks, including, PGD [4], NI-FGSM [11], and Diff-PGD [8] and AdaMSI-FGM [16]. Our focus is on transfer-based attacks, where no query access to the target model is granted. Therefore, we do not compare with query-based methods.

**Evaluation metrics.** We evaluate the performance using the standard evaluation metric for adversarial attacks, Attack Success Rate (ASR), which is used to assess the quality of the adversarial example. ASR is defined as the percentage of adversarial examples that are misclassified by the target model. Higher ASR values indicate better adversarial example quality. Additionally, we leverage Frechet Inception Distance (FID) [54] as the indicator of the human imperceptibility of the crafted adversarial examples. A full-referenced metric, LPIPS [55], is also used to assess the perceptual differences.

**Implementation details.** Our experiments were conducted using an NVIDIA L20 GPU. Some experiments follow [56] to support the state-of-the-art baselines, and Diff-PGD follow [8]. The software versions used are Ubuntu 22.04.5, Python 3.11.11, PyTorch 2.5.1+cu124, and Torchvision 0.20.1. Previous research indicates that more iterations are favorable for convergence [57]. In order to better demonstrate the convergence process, *n* = 20 is adopted in our experiments. The maximum of $L_{\infty }$ norm perturbation ϵ=4/255, and the batch size is set to 64 for all algorithms. For MI-FGSM and NI-FGSM, we adopt the default momentum parameter μ=1 and step-size $\alpha_{n}=4/255/20$. For PGD, Diff-PGD, and AdaMSI-FGM, the step-size $\alpha_{n}=4/255/20$. The remaining hyperparameters of AdaMSI-FGM were set to their default values, following the original paper [16]. For Diff-AdaNAG, we set η=0.05, $\theta_{0}=1$ and δ=1e−16.

### Results of adversarial attacks

In this section, we report various experimental results to demonstrate the effectiveness of the proposed Diff-AdaNAG method. First, we compare the performance of Diff-AdaNAG with several classic black-box and white-box attacks. Then, we compare the convergence between the proposed method and existing methods. Furthermore, we show the flexibility of the proposed method by combining Diff-AdaNAG with other existing black-box attacks such as Diverse Input Method (DIM) [58], Translation-Invariant Method (TIM) [59], and Scale-Invariant Method (SIM) [11]. Additionally, we also conduct an in-depth comparison with SI-NI-FGSM and several advanced feature-level attacks. Finally, we visualize the generated adversarial examples to demonstrate the stealthiness of the proposed method.

#### Comparison with classic attacks.

We compare the performance of Diff-AdaNAG with the classic attacks. The ASRs against the considered models are shown in Table 1.

**Table 1:** Attack success rates (%) of adversarial attacks against twelve models. ^*^ indicates the white-box attacks.

Model	Attack	Res-18	Res-34	Res-50	Res-101	EffNet	GooLeNet	Inc-v3	MNAS	MobNet	ShufNet	SqueNet	VGG-11	FID ↓	LPIPS ↓
Res-18	PGD	99.8 ^*^	46.4	41.2	35.6	33.6	38.2	38.4	43	40.4	48.6	51.6	47.6	**16.12**	**0.03**
	Diff-PGD	99.8 ^*^	63.4	60.2	53	45.8	54.4	48.6	54.4	47	56	63.2	62.4	33.3	0.05
	AdaMSI-FGM	99.8 ^*^	63.4	54.4	50.8	44.4	50.2	45	51.2	45.2	54.8	61.8	58.4	28.4	0.06
	NI-FGSM	**100** ^*^	72.8	63	57	49	54.8	48	57	47.4	57.2	65.8	64.2	36.03	0.06
	Diff-AdaNAG	99.8 ^*^	**79.4**	**67**	**62.6**	**53.6**	**61**	**51.8**	**59.4**	**52.2**	**59.2**	**70.4**	**68.6**	23.68	**0.03**
Res-34	PGD	51	**100** ^*^	40.6	36.6	33.2	38.2	37.4	41.2	39.8	48	51.2	43.4	**16.1**	**0.03**
	Diff-PGD	73.6	**100** ^*^	64	55.8	46.8	51.4	48.2	53	45	54.4	63.6	59	34.73	0.05
	AdaMSI-FGM	65.4	**100** ^*^	59.4	53.6	43.4	50.2	46.6	51.6	43.6	52	58.2	54.4	29.84	0.06
	NI-FGSM	75.2	**100** ^*^	68.2	62	51.4	53.4	48.6	54.2	46.2	**56**	63.2	60	36.94	0.06
	Diff-AdaNAG	**79.6**	**100** ^*^	**70.4**	**66.2**	**54.2**	**60.2**	**54.4**	**57.8**	**48.2**	55.2	**67**	**65.4**	24.92	**0.03**
Res-50	PGD	44.8	41	99.4^*^	40.2	34.2	37.4	37.8	40	39.8	48.2	49.6	41.8	**13.56**	**0.02**
	Diff-PGD	64.8	64.4	99.6 ^*^	69	45.8	50	47	49.8	44.8	51.6	56.6	55.8	33.64	0.05
	AdaMSI-FGM	59.2	56.4	99.6 ^*^	60	44.4	46.6	45	46.4	43.6	51	56.8	52.8	27.91	0.05
	NI-FGSM	64	63.2	**99.8** ^*^	71	48	50	47	51.8	44.6	53.8	58.8	57.6	33.83	0.05
	Diff-AdaNAG	**70.4**	**71**	99.6 ^*^	**79**	**54**	**55.6**	**51.4**	**54.2**	**47**	**55**	**59.8**	**61.6**	23.97	0.03
Res-101	PGD	44.6	42	43.6	**100** ^*^	34	37.6	38	40.2	39.4	46.6	48.4	41.2	**13.64**	**0.02**
	Diff-PGD	61	59.8	70.8	**100** ^*^	45.6	47.6	46.8	47.4	44.4	49.8	55.4	52.2	33.52	0.05
	AdaMSI-FGM	58	54.4	65.4	**100** ^*^	44.8	47.6	45.2	46	42.8	51.2	54.2	50.8	27.41	0.05
	NI-FGSM	62	62.4	74.2	**100** ^*^	48.2	50.8	48.2	51	45	**54.2**	56.8	54.6	32.94	0.05
	Diff-AdaNAG	**67**	**68.6**	**77.6**	99.8 ^*^	**53.2**	**54.8**	**50.8**	**52.2**	**47.6**	52.6	**59**	**58.8**	23.67	0.03
EffNet	PGD	44.8	41	38.6	36.2	99.2^*^	37.8	42	50	44.6	49.4	51.8	46	**20.42**	**0.03**
	Diff-PGD	57.4	51	48.8	48.8	99.6 ^*^	49.6	49.2	64.4	56	55	59.6	57.6	39.94	0.06
	AdaMSI-FGM	55	50.4	53.6	47.2	**100** ^*^	45	48.8	60.6	53.6	55.6	59	55.6	34.62	0.06
	NI-FGSM	58.2	55	53.6	49.4	**100** ^*^	48	51.4	64.4	57.8	57.2	61	58.8	39.48	0.06
	Diff-AdaNAG	**62.2**	**56.8**	**56.8**	**53.2**	99.4^*^	**52.8**	**53.2**	**70.2**	**62.6**	**59**	**62.6**	**63**	29.55	**0.03**
GooLeNet	PGD	40.2	36	33.6	31	31.8	**100** ^*^	38.6	39.4	39.2	48	49.4	40.2	**13.45**	**0.02**
	Diff-PGD	52.8	47.2	44.4	40	40	**100** ^*^	47.8	48	44.8	51.6	56.8	51.2	31.6	0.05
	AdaMSI-FGM	51.2	47.4	42.4	38.8	40	**100** ^*^	46.8	47.6	43.8	50.8	56.85	50.6	27.66	0.05
	NI-FGSM	54.2	49	47	41.2	42.6	**100** ^*^	48.6	48.2	45.4	**52.8**	59	53.2	31.66	0.05
	Diff-AdaNAG	**58.8**	**53.4**	**51.8**	**45**	**46**	**100** ^*^	**54**	**53.2**	**48.2**	**52.8**	**59.8**	**57.4**	23.05	0.03
Inc-v3	PGD	38.8	35	30.6	29.6	30.2	35	98.4^*^	38.6	38.2	46.8	45.2	38.2	**11.53**	**0.02**
	Diff-PGD	45.6	42.6	36.6	34.6	36.2	43	**99** ^*^	41.6	41.4	49.2	50.4	43.6	26.31	0.04
	AdaMSI-FGM	44.4	40.6	38.6	36.6	36	42.2	98.8 ^*^	43.6	41	49.2	50.2	44	25.32	0.05
	NI-FGSM	46.2	44	38.6	37.6	37.6	43.4	**99** ^*^	44.2	41.4	**50.6**	50.4	43.6	29.47	0.05
	Diff-AdaNAG	**47.4**	**45.4**	**39.6**	**38.8**	**38.8**	**45.6**	98^*^	**45**	**42.6**	50	**51.4**	**45.4**	20.11	0.03
MNAS	PGD	38.8	33.4	31.6	29	34	35	34.6	**100** ^*^	43.4	48.8	50.4	41.2	**9.84**	**0.02**
	Diff-PGD	45.4	41.2	37.4	34.8	42.6	39.6	40.2	**100** ^*^	55.4	53.6	58	49.2	24.11	0.05
	AdaMSI-FGM	45.4	42.6	37.8	34.2	42.6	40.2	40.8	**100** ^*^	53.4	54.8	58.2	49.2	20.84	0.05
	NI-FGSM	47.8	43.4	40.2	35	45.8	41.8	41.4	**100** ^*^	56.8	55	60.8	52.2	23.77	0.05
	Diff-AdaNAG	**50.4**	**45.4**	**40.4**	**37.4**	**50.4**	**44.8**	**42.2**	**100** ^*^	**62**	**57.6**	**62.4**	**54.2**	19.74	0.03
MobNet	PGD	38.6	33.8	31	29	34.4	34	34.8	49	**100** ^*^	49	51	41	**11.3**	**0.02**
	Diff-PGD	43.6	39.4	37	34.2	44.2	39.6	42.2	63.4	**100** ^*^	54.4	55.8	47.6	23.11	0.05
	AdaMSI-FGM	43.8	40	36	33.6	42.2	39.4	41.8	59	**100** ^*^	53.8	56.4	46.8	21.07	0.05
	NI-FGSM	45.4	40.4	38.2	35.2	44.6	41.2	41.4	64.6	**100** ^*^	55.2	55.4	49.2	23.81	0.05
	Diff-AdaNAG	**46.8**	**42.6**	**39.4**	**35.6**	**48.6**	**42.6**	**44**	**71.4**	**100** ^*^	**57.6**	**59.4**	**52.6**	21.56	0.03
ShufNet	PGD	38.2	33.6	29	28.2	30.6	34	34	40.8	39.8	**100** ^*^	49.4	37.6	**8.91**	**0.02**
	Diff-PGD	45.6	38.4	34.8	32.6	34.4	37.6	38	48.8	43.8	**100** ^*^	57.4	44.2	16.91	0.05
	AdaMSI-FGM	44.2	37.6	34.8	32	33	36.8	38	47.8	43.4	**100** ^*^	55.8	42.6	16.78	0.06
	NI-FGSM	45	39.4	34.4	32	35	38.4	35.8	49.6	44.6	**100** ^*^	57.2	43.2	17.4	0.05
	Diff-AdaNAG	**48**	**40.6**	**36.6**	**35.2**	**37.6**	**38.6**	**39.2**	**52**	**46.2**	**100** ^*^	**60.6**	**46.8**	12.86	0.03
SqueNet	PGD	40.8	35.6	31.8	29.2	31	34.8	34.4	40.2	40	50.4	**100** ^*^	43	**12**	0.12
	Diff-PGD	48.8	41.4	35	33.6	35.6	42.8	39	49.2	44	54.6	**100** ^*^	52	21.36	0.14
	AdaMSI-FGM	47.4	41.4	36.2	34.4	36	42.2	38.8	46.4	44.2	55	**100** ^*^	50.8	20.64	0.14
	NI-FGSM	50	43.2	37.8	34.8	36.6	42.4	38.2	50.6	44.2	56.6	**100** ^*^	52.8	22.62	0.16
	Diff-AdaNAG	**54.6**	**45.2**	**39**	**36.4**	**40.2**	**45.8**	**40.8**	**54.2**	**46.6**	**57.8**	**100** ^*^	**56.6**	16.28	**0.07**
VGG-11	PGD	45	39.6	36.2	32.6	35.4	37.2	37.6	42.6	41.2	48.2	52.6	99.8 ^*^	**21.68**	**0.03**
	Diff-PGD	65.2	57.4	50.4	43	47.2	51.4	46.4	55.6	49.4	54.4	65.2	99.8 ^*^	38.15	0.05
	AdaMSI-FGM	59.4	52.4	48.8	42.8	46.4	45.2	44	54.2	45.6	53	63.6	**100** ^*^	32.7	0.05
	NI-FGSM	63.2	55.6	53.8	47	51.2	49.2	45.2	57	47.6	54.6	66	**100** ^*^	38.7	0.05
	Diff-AdaNAG	**70.8**	**63**	**59**	**50.6**	**56.6**	**55.6**	**49.6**	**63.4**	**50.4**	**57.8**	**72.8**	99.8 ^*^	28.04	**0.03**

The best result is bolded, and the second-best result is underlined.

It’s not hard to see from Table 1 that all algorithms exhibit strong white-box attack performance, with nearly 100% ASRs against all white-box models. On the other hand, our Diff-AdaNAG outperforms all other methods in black-box attacks by integrating AdaNAG and diffusion-based schemes.

#### Convergence comparison with diff-PGD.

**Fig 2:**
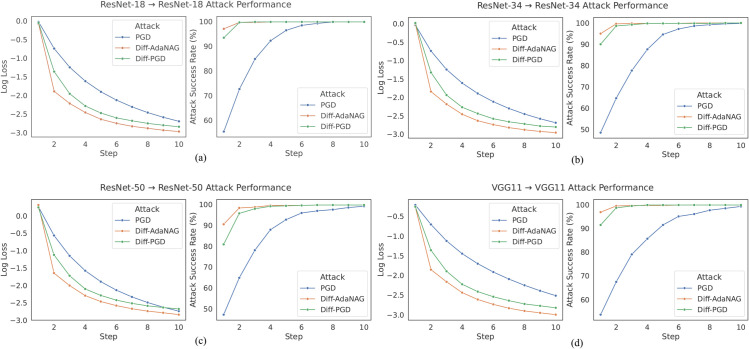
Logarithm of loss and attack success rates (%) of Diff-AdaNAG, Diff-PGD, and PGD on various numbers of iterations. The adversarial examples are generated on ResNet-18 (a), ResNet-34 (b), ResNet-50 (c), and VGG11 (d). The x-axis represents the number of iterations, and the y-axis represents the attack success rates and the logarithm of loss.

To compare the convergence of the proposed Diff-AdaNAG, we use Diff-AdaNAG, Diff-PGD, and PGD to generate the adversarial examples on ResNet-18, ResNet-34, and ResNet-50, and VGG11. Specifically, the adversarial examples are crafted on the models with various numbers of iterations ranging from 1 to 10, and we log the attack success rates (ASR) of the generated adversarial examples and loss values at each iteration. The results are shown in Fig 2. It can be observed that Diff-PGD converges faster than PGD, which is consistent with the results in [8] and verifies the effectiveness of introducing the diffusion mechanism. Furthermore, our Diff-AdaNAG achieves the best convergence performance among all methods, and it yields higher attack success rates than other methods with the same number of iterations. In another view, Diff-AdaNAG achieves the same attack success rates as other methods with fewer iterations. This indicates that the proposed method can achieve a better convergence performance by combining the diffusion-based scheme with NAG.

#### Flexibility

To enhance the flexibility and transferability of our proposed method, we incorporate mechanisms from several existing black-box attack techniques. Specifically, we explore combinations of our method (Diff-AdaNAG) with three representative approaches: DIM [58], TIM [59], and SIM [11]. In our experiments, the baseline is an iterative FGSM variant (namely BIM or I-FGSM). We systematically denote the integrated variants—for instance, “DI” represents DIM combined with I-FGSM, whereas “DI+Ours” indicates DIM integrated with our proposed method. We conduct extensive experiments to evaluate both individual and joint combinations of these methods. In addition to assessing DI, TI, and SI separately, we also experiment with pairwise combinations such as DI+SI, DI+TI, and SI+TI, as well as the full combination DI+TI+SI.

From Table 2, it is evident that our method, when combined with DIM (denoted DI+Ours) and SIM (denoted SI+Ours), consistently outperforms their corresponding baselines (DI and SI alone). Specifically, DI+Ours and SI+Ours demonstrate clearly higher attack success rates across various model combinations. This indicates that our method has strong compatibility and synergy with DIM and SIM. However, when our method is integrated with TIM (denoted TI+Ours), although performance improves compared to baseline (TI alone), it remains inferior to our method without TI (see Table 1). The reason could be that TIM inherently averages gradients over spatial translations, potentially diminishing the strength of location-sensitive adversarial features generated by our method. Consequently, while TI enhances translation invariance, it may weaken essential high-frequency perturbations required for successful cross-model attacks, thus leading to comparatively lower performance. The results of other source models (MNASNet, MobileNet, etc.) are provided in S1 Table,S2 Table, and S3 Table.

**Table 2:** Attack success rates (%) of adversarial attacks against twelve models. ^*^ indicates the white-box attacks.

Comparison of DI and DI+Ours													
Model	Attack	Res-18	Res-34	Res-50	Res-101	EffNet	GooLeNet	Inc-v3	MNAS	MobNet	ShufNet	SqueNet	VGG-11
Res-18	DI	**99.8**	64.8	56.8	48.6	43.6	52.2	46.4	53	45	53.4	61	59.6
	DI+Ours	**99.8**	**81.2**	**69.8**	**66**	**57**	**68.2**	**61.2**	**65.8**	**54.2**	**61.8**	**75**	**72.4**
Res-34	DI	70.6	**100**	58.8	54	43.4	52	47	52.2	45	52	59.2	54.2
	DI+Ours	**85.4**	**100**	**74.8**	**71**	**58.2**	**66.8**	**62**	**62.6**	**50.8**	**59.2**	**69.4**	**68**
Res-50	DI	60.4	56.4	**99.8**	59.4	45.2	49.8	45.6	47.8	44.8	51	54.8	52.8
	DI+Ours	**76.6**	**75**	99.4	**80.6**	**60.2**	**65.4**	**59.8**	**59**	**51.4**	**56.6**	**64.8**	**66.2**
Res-101	DI	59.4	56.2	67.2	**100**	45.4	47.6	45.6	47.2	42.4	50	53.2	52
	DI+Ours	**73.6**	**75.4**	**82.2**	99.6	**59.4**	**64.8**	**61**	**57**	**50.6**	**57**	**63.8**	**64**
**Comparison of SI and SI+Ours**													
**Model**	**Attack**	**Res-18**	**Res-34**	**Res-50**	**Res-101**	**EffNet**	**GooLeNet**	**Inc-v3**	**MNAS**	**MobNet**	**ShufNet**	**SqueNet**	**VGG-11**
Res-18	SI	**100** ^*^	69.2	58.2	53.8	45.6	53.6	46.8	53.4	46.2	55.4	65	61.6
	SI+Ours	**100** ^*^	**81.8**	**69.8**	**62.8**	**56.6**	**66.8**	**57.4**	**63**	**53.2**	**61.6**	**75**	**71**
Res-34	DI	70.4	**100** ^*^	60.4	52.8	43.4	51	47	50	43.6	52.6	60.2	56.6
	DI+Ours	**85.6**	99.8^*^	**73.8**	**68.4**	**55.4**	**65.8**	**59.2**	**60.4**	**50**	**59.4**	**69.8**	**66**
Res-50	SI	59.6	58.6	**99.2** ^*^	61.8	41.8	49	45.6	46.6	43	52.2	56	53.6
	SI+Ours	**76.6**	**75.8**	98.8^*^	**79.4**	**54.2**	**65**	**56.8**	**57**	**51**	**57**	**64.2**	**64.8**
Res-101	SI	60.4	58.2	68.4	**99** ^*^	44.2	49.6	45.2	46.8	43	50.6	55	50.6
	SI+Ours	**73.2**	**75.4**	**82.2**	98.6^*^	**56**	**65.4**	**60.2**	**55.8**	**49.4**	**56.6**	**63.8**	**61.6**
**Comparison of TI and TI+Ours**													
**Model**	**Attack**	**Res-18**	**Res-34**	**Res-50**	**Res-101**	**EffNet**	**GooLeNet**	**Inc-v3**	**MNAS**	**MobNet**	**ShufNet**	**SqueNet**	**VGG-11**
Res-18	TI	**99.8** ^*^	51.8	45.6	41.2	36.8	41.2	40.8	51.2	46.2	49.8	55.2	50.8
	TI+Ours	**99.8** ^*^	**64.4**	**52**	**46.6**	**40.6**	**46.8**	**46**	**58.4**	**51.2**	**53**	**61.8**	**58.4**
Res-34	TI	56.6	**100** ^*^	45	41.6	36	42.8	42	47.8	44.2	49.6	53.6	47.8
	TI+Ours	**67.8**	99.8^*^	**55.4**	**48.4**	**40.8**	**46.6**	**46.6**	**55.4**	**50.4**	**52**	**57.4**	**53.8**
Res-50	TI	50	48	**99.6** ^*^	45.8	35.2	40.4	39.6	44.6	43.2	48.2	51.2	45.6
	TI+Ours	**59.2**	**58.4**	99.2^*^	**59.2**	**42.8**	**46.8**	**46.2**	**52.4**	**50.4**	**51**	**55**	**54**
Res-101	TI	48.2	45	51	**100** ^*^	36.4	40.2	39.2	44	44.4	47.8	51	44
	TI+Ours	**56.8**	**56**	**63.6**	99.4^*^	**44**	**46.6**	**47.2**	**51.4**	**48.4**	**51.8**	**53.6**	**50.8**

The best result is bolded.

To further verify that the performance degradation of TI stems from its intrinsic issues, we conducted experiments on pairwise combinations of TI, DI, and SI, with results presented in Table 3. Our experiments show that combining DI and SI with our method (DI+SI+Ours) achieves a significantly higher attack success rate than using DI, SI, or our method alone, indicating strong synergy between our method and DI/SI. However, when any method is combined with TI, the performance drops significantly, even below the baselines (e.g., DI+TI underperforms DI, and SI+TI underperforms SI). This confirms that TI’s inherent inflexibility negatively impacts performance. In contrast, our method is plug-and-play and consistently outperforms baselines across all scenarios, demonstrating the superior transferability and flexibility of Diff-AdaNAG. The results of other source models (MNASNet, MobileNet, etc.) are provided in S4 Table,S5 Table, and S6 Table.

**Table 3:** Attack success rates (%) of adversarial attacks against twelve models. ^*^ indicates the white-box attacks.

Comparison of DI+SI and DI+SI+Ours													
Model	Attack	Res-18	Res-34	Res-50	Res-101	EffNet	GooLeNet	Inc-v3	MNAS	MobNet	ShufNet	SqueNet	VGG-11
Res-18	DI+SI	99.4^*^	75.4	63.2	58	49.2	62.6	53.6	58.4	49.4	59.8	70.4	66.6
	DI+SI+Ours	**100** ^*^	**85.6**	**74.4**	**67.6**	**60.4**	**76.6**	**67.4**	**69.4**	**57.2**	**64.8**	**78**	**76.2**
Res-34	DI+SI	77.6	99.6^*^	66	60.6	50	61.6	55	56	48.2	56.2	65.6	61.8
	DI+SI+Ours	**87.6**	**99.8** ^*^	**78**	**74.6**	**60.6**	**73.2**	**66.8**	**66.6**	**54.2**	**62.4**	**72.8**	**70.8**
Res-50	DI+SI	67.4	65.6	95.6^*^	66	49	59	54.6	53.8	46.8	54.8	58.8	58.4
	DI+SI+Ours	**82**	**80.4**	**98.6** ^*^	**84.2**	**62.8**	**72.8**	**67.8**	**64.2**	**55**	**60.6**	**69.8**	**69.8**
Res-101	DI+SI	67.8	66.6	73.4	97.6^*^	51.4	58	52.8	51.6	48.4	53.8	59.2	58.4
	DI+SI+Ours	**79.2**	**81.2**	**84.4**	**98.4** ^*^	**64**	**71.6**	**68.6**	**63.6**	**53.6**	**60.4**	**69.6**	**66.8**
**Comparison of DI+TI and DI+TI+Ours**													
**Model**	**Attack**	**Res-18**	**Res-34**	**Res-50**	**Res-101**	**EffNet**	**GooLeNet**	**Inc-v3**	**MNAS**	**MobNet**	**ShufNet**	**SqueNet**	**VGG-11**
Res-18	DI+TI	**99.8** ^*^	51.4	44.8	40.8	37.2	42.4	41.2	50.8	45.8	50.2	55.4	50.6
	DI+TI+Ours	99.6^*^	**64.4**	**54.6**	**46.4**	**42.8**	**51.2**	**49.4**	**59.6**	**52.8**	**53.8**	**63.6**	**60.6**
Res-34	DI+TI	58.8	**100** ^*^	47.4	43	37.6	43.6	43.6	49.2	45.6	49.4	53.4	48.4
	DI+TI+Ours	**71.8**	**100** ^*^	**56.2**	**51**	**43**	**50.4**	**50.2**	**56.6**	**51.2**	**53.4**	**60.6**	**55.6**
Res-50	DI+TI	51.6	49.8	98.4^*^	46.6	37.6	43.2	41.4	47.6	45.4	48	52.8	47.8
	DI+TI+Ours	**64.6**	**61.2**	**99.2** ^*^	**59.6**	**44.8**	**50**	**49.2**	**57.8**	**52.8**	**52.6**	**58.2**	**56**
Res-101	DI+TI	51.8	48.6	50.8	98.2^*^	39	42	40.6	45.4	44.2	48.2	50.4	45.4
	DI+TI+Ours	**62.6**	**59**	**67.2**	**99** ^*^	**46.6**	**50.8**	**47**	**55**	**51.2**	**54.4**	**55.8**	**54**
**Comparison of SI+TI and SI+TI+Ours**													
**Model**	**Attack**	**Res-18**	**Res-34**	**Res-50**	**Res-101**	**EffNet**	**GooLeNet**	**Inc-v3**	**48**	**MobNet**	**ShufNet**	**SqueNet**	**VGG-11**
Res-18	SI+TI	95.6^*^	56.8	48.2	42.6	40	46	43.8	53.8	50	51	59.2	55.4
	SI+TI+Ours	**97.8** ^*^	**63.6**	**52.4**	**45.6**	**42.2**	**51.4**	**48**	**60**	**55.2**	**53.4**	**63.8**	**59.2**
Res-34	SI+TI	63.4	93.6^*^	51.4	45.2	38.8	45.8	46.4	51.4	47	50.4	55.2	52
	SI+TI+Ours	**69.6**	**97.8** ^*^	**56.4**	**48.6**	**41.6**	**50.8**	**48.8**	**57.4**	**53.4**	**53.6**	**59**	**55**
Res-50	SI+TI	55.8	53	90.6^*^	51.8	39.8	46.8	43	50.8	48.2	50.2	54	50
	SI+TI+Ours	**63.6**	**60.4**	**94.2** ^*^	**58.6**	**43.4**	**51.8**	**50.8**	**57.4**	**53.4**	**55.6**	**58**	**55.2**
Res-101	SI+TI	53.2	49.6	55.2	89^*^	41.2	44	43.8	48.2	47.8	50.2	52.4	48.2
	SI+TI+Ours	**60.6**	**58.8**	**63.6**	**93.4** ^*^	**44**	**49.2**	**48.4**	**54.4**	**51.2**	**52.8**	**56.2**	**53.2**

The best result is bolded.

When extending the analysis to combinations involving all three transformations (SI+TI+DI), the experimental results align with the above observations. As shown in Table 4, SI+TI+DI outperforms SI+TI and DI+TI but underperforms DI+SI, further confirming that TI detracts from overall attack success rates when combined with other methods. Nevertheless, incorporating our method (SI+TI+DI+Ours) significantly enhances the baseline combination (SI+TI+DI), boosting its transferability. The results of other source models (MNASNet, MobileNet, etc.) are provided in S7 Table.

**Table 4:** Attack success rates (%) of adversarial attacks against twelve models. ^*^ indicates the white-box attacks.

Model	Attack	Res-18	Res-34	Res-50	Res-101	EffNet	GooLeNet	Inc-v3	MNAS	MobNet	ShufNet	SqueNet	VGG-11
Res-18	SI+TI+DI	93.4^*^	57	47.8	42.4	39.8	49	45.4	56.6	50.4	51.8	60	55.2
	SI+TI+DI+Ours	**97.2** ^*^	**66**	**53.8**	**46.6**	**45**	**55.2**	**50.8**	**61.6**	**57.6**	**56.2**	**65.4**	**61**
Res-34	SI+TI+DI	62.8	90.2^*^	50.2	45.6	38.2	48.6	48	53	48.4	51.2	56.4	52.4
	SI+TI+DI+Ours	**73.6**	**96.4** ^*^	**57.4**	**50**	**42.8**	**54**	**52.8**	**59.8**	**55**	**53.6**	**63.2**	**57.8**
Res-50	SI+TI+DI	58	55	86	51.6	40	48.4	45.8	51	49	52.2	55.8	51.4
	SI+TI+DI+Ours	**67**	**62.4**	**93.2** ^*^	**59**	**45.4**	**54.6**	**53.4**	**60**	**54.4**	**57.2**	**60.8**	**56.6**
Res-101	SI+TI+DI	56.2	52.2	55.6	85^*^	41.2	46.2	44.6	49.6	48	51.8	54.4	49
	SI+TI+DI+Ours	**64.4**	**59.8**	**66.2**	**91.2** ^*^	**46.4**	**54.6**	**52.6**	**56.6**	**53.8**	**54.8**	**59.2**	**53.8**

The best result is bolded.

#### Further comparison with NI-FGSM.

As stated in the first comparison of this section, our method outperforms all compared algorithms, including NI-FGSM, in both white-box and black-box scenarios. To further highlight the performance gap between our method and NI-FGSM, we conduct a deeper comparison. Reference [11] introduced two adversarial attack methods, NI-FGSM and SIM, and demonstrated that SIM can be combined with other attack methods to enhance performance. This conclusion is also validated in the subsection above (e.g., SI+DI outperforms DI, SI+TI outperforms TI, and SI+TI+DI outperforms TI+DI). Reference [11] further emphasizes that combining SIM with NI-FGSM to form the SI-NI-FGSM method significantly improves attack performance, particularly in challenging black-box scenarios. Therefore, we compare our method with SI-NI-FGSM by introducing SI to form SI-DiffAdaNAG (SI+Ours). The adversarial examples are crafted on ResNet-18 with various numbers of iterations ranging from 0 to 20 and then transferred to attack ResNet-34, ResNet-50, and ResNet-101.

**Fig 3:**
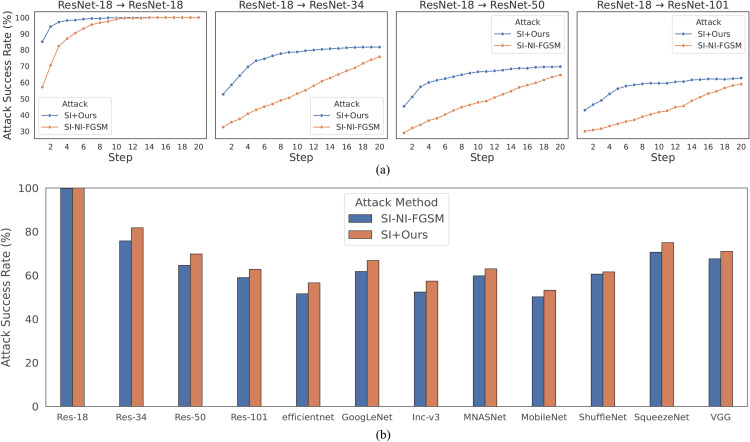
Comparison of SI-NI-FGSM and SI+Ours. (a) Attack success rates of different model combinations with increasing steps, attacks are launched from ResNet-18 and transferred to four target models: ResNet-18 (white-box), ResNet-34, ResNet-50, and ResNet-101 (black-box settings); (b) Attack success rates of different models, the source model is ResNet-18.

As shown in Fig 3(a), SIM, combined with our proposed method (denoted as SI+Ours), consistently achieves superior attack success rates compared to the original SI-NI-FGSM across different ResNet model combinations. Specifically, when attacking ResNet-18 itself, both methods show rapidly increasing success rates with more steps. However, SI+Ours reaches near-perfect success faster, indicating stronger attack capability. When the adversarial examples generated by ResNet-18 are transferred to black-box settings by choosing ResNet-34, ResNet-50, and ResNet-101 as the target models, our method consistently achieves higher success rates than SI-NI-FGSM as the step count increases, demonstrating improved transferability and robustness. Notably, at step 20, the attack success rate of SI+Ours exceeds SI-NI-FGSM by approximately 5%, highlighting a substantial improvement in the transferability.

Additionally, the comparative results in Fig 3(b) further reinforce the effectiveness of our enhanced method across a variety of architectures, including ResNet series, EfficientNet, GoogLeNet, Inception-v3, and other models described in the experimental setting. Across all tested architectures, SI+Ours consistently surpasses the baseline SI-NI-FGSM, with improvements ranging from approximately 3% to 10% in terms of attack success rates. This consistent improvement across diverse architectures demonstrates the generalization ability of our method in black-box transfer settings. The results for the remaining models (MNASNet, MobileNet, etc.) are provided in S2 Table.

Overall, the performance divide between NI-FGSM and Diff-AdaNAG further illustrates the gap between NAG method in optimization theory and its adversarial attack practice. The results not only indicate that Diff-AdaNAG has a better transferability, but also demonstrate that with the property of looking ahead, Diff-AdaNAG can accelerate the generation of adversarial examples, making it highly effective for generating transferable adversarial attacks in practical scenarios.

#### Further comparison with advanced feature-level attacks.

In this subsection, we compare our approach with several advanced feature-level attacks that incorporate additional techniques, such as exploiting information extracted from intermediate layers of classifiers (FIA [18], NEAA [19], MFAA [20]), as well as leveraging channel redundancy and invariance to perturb convolutional features (CAAM [21]).

It should be noted that all comparative experiments in this paper were conducted with a perturbation budget of 4/255, rather than the 16/255 adopted in the original papers. The attack models evaluated in this subsection include ResNet-152 (Res-152) [44], Inception-v3 (Inc-v3) [47], Inception-v4 (Inc-v4) and InceptionResNet-v2 (IncRes-v2) [60], as well as VGG16 (VGG-16) and VGG19 (VGG-19) [52]. For FIA, NEAA, and MFAA, we adopt the attack layers of Mixed_5b for Inc-v3, Mixed_5a for Inc-v4, conv2d_4a for IncRes-v2, Conv3_3 for VGG-16, Conv3_4 for VGG-19, and the final layer of the second block for Res-152, respectively. For MFAA, since it requires fusing multiple layers of feature representations, in addition to the designated attack layer we further select supplementary layers. Specifically, for Res-152 we follow the configuration provided in its official GitHub repository (https://github.com/KWPCCC/MFAA) and include unit 9 of the third block (layer3.8), unit 19 of the third block (layer3.18), unit 29 of the third block (layer3.28), and the final convolutional layer (layer4), which we summarize as [layer2, layer3.8, layer3.18, layer3.28, layer4]. For the remaining source models, where the original paper does not explicitly specify additional layers, we adopt a similar strategy to that of Res-152. Concretely, we use [Mixed_5b, Mixed_5d, Mixed_6e, Mixed_7c] for Inc-v3; [Mixed_5a, ReductionA, ReductionB, InceptionC] for In-v4; [conv2d_4a, mixed_6a, mixed_7a, conv2d_7b] for IncRes-v2; [conv3_3, conv4_3, conv5_3] for VGG-16; and [conv3_4, conv4_4, conv5_4] for VGG-19. All other implementation details are kept consistent with those reported in the Implementation Details of Experiments sections. The ASRs against the considered six models are shown in Table 5.

**Table 5:** Attack success rates (%) of adversarial attacks against six models. ^*^ indicates the white-box attacks.

Model	Attack	Inc-v3	Inc-v4	IncRes-v2	VGG-16	VGG-19	Res-152	FID ↓	LPIPS ↓
Inc-v3	FIA	95^*^	**59.4**	50	**46.4**	**46.4**	**40.6**	54.57	0.06
	NEAA	95^*^	56	48.8	45.8	45.8	37.8	50.6	0.06
	MFAA	95.6 ^*^	57.2	**52**	45	45	39.8	54.47	0.06
	CAAM	85.8^*^	53.6	51	43.6	43	39.2	33.17	0.05
	Diff-AdaNAG	**98.2** ^*^	51	48.6	42.6	42.6	36.4	**20.11**	**0.03**
Inc-v4	FIA	53.2	95.2^*^	**51**	46.6	46.6	37.6	48.47	0.06
	NEAA	46.8	96 ^*^	44.4	43.8	43.8	33.8	44.03	0.06
	MFAA	53.8	**98** ^*^	49.6	**47.2**	**47.2**	**38**	50.83	0.06
	CAAM	**54.4**	81.2^*^	49.8	45.8	44.2	37.8	32.67	0.05
	Diff-AdaNAG	51.2	95.2^*^	45.4	42	42	34.6	**17.5**	**0.02**
IncRes-v2	FIA	**53.6**	**57.2**	89^*^	42.8	42.8	33.2	42.15	0.06
	NEAA	**51.2**	53.6	88.8^*^	**43.6**	**43.6**	33.4	42.57	0.06
	MFAA	49	50.2	**90** ^*^	40.8	40.8	31.6	39.87	0.06
	CAAM	48.4	49	67.2^*^	39.2	40.4	33.4	25.95	0.05
	Diff-AdaNAG	49.2	53.2	89.8 ^*^	41.6	41.6	**35.6**	**17.59**	**0.03**
VGG-16	FIA	42.4	46.4	40	98.4^*^	77.6	37.6	62.77	0.07
	NEAA	41.2	44.4	37.6	95.4^*^	68.4	33.4	53.63	0.07
	MFAA	40.4	44.2	39.4	95^*^	70.8	36	58.87	0.07
	CAAM	**51.6**	**54**	**46**	99 ^*^	91.4	**47.2**	52.75	0.06
	Diff-AdaNAG	47.2	50.4	42.2	**99.2** ^*^	**93.2**	43.6	**24.53**	**0.03**
VGG-19	FIA	43.2	49	39	83.4	**99.6** ^*^	38	65.5	0.07
	NEAA	41.4	45.2	38.2	75.4	97.4^*^	33.2	57.74	0.07
	MFAA	42.2	46.6	39	78.4	98.2^*^	37	61.53	0.07
	CAAM	**51.4**	53	**47.2**	89.8	99^*^	**47**	53.24	0.06
	Diff-AdaNAG	47.6	**53.4**	43.8	**92.8**	99.2 ^*^	46.8	**25.7**	**0.03**
Res-152	FIA	51.4	52.2	45.4	53.2	53.2	95.4 ^*^	62.24	0.06
	NEAA	46	47.4	42.8	48.2	48.2	92^*^	53.79	0.06
	MFAA	48.8	50	44.8	52.2	52.2	93^*^	61.77	0.06
	CAAM	**57.6**	**56.2**	**50.8**	58.2	58	94.4^*^	46.54	0.06
	Diff-AdaNAG	53.4	52.4	48	**59.8**	**59.8**	**99.4** ^*^	**24.93**	**0.03**

The best result is bolded, and the second-best result is underlined.

The results show that in the white-box scenario, the proposed method consistently pushes the success rate to over 99% on VGG-16/19 and Res-152, and to 98.2% on Inc-v3, outperforming the second-best method by 2–14 absolute points. More importantly, our method also obtains the highest average black-box ASR (45.0%), surpassing MFAA (43.9%) and CAAM (42.8%) while leaving FIA/NEAA further behind (≈ 41%). The improvement is especially pronounced when Res-152 or either VGG model serves as the surrogate, yielding cross-model gains of +2.6% and +4.6% over the runner-up, respectively.

#### Human-imperceptible.

**Quantitative analysis.** In the last two columns of Tables 1 and 5, we report FID and LPIPS, where the former serves as an indicator of the human imperceptibility of the crafted adversarial examples, and the latter is used to assess their perceptual differences. From the results of Table 1, we can observe that our method outperforms the others on both metrics except for PGD. Although PGD achieves slightly better imperceptibility scores, this may stem from its weaker attack strength, as reflected in its inferior ASR performance. By contrast, our method consistently achieves higher ASR while maintaining favorable perceptual quality. From the results of Table 5, Diff-AdaNAG exhibits the smallest perceptual distortion, achieving an average FID of 20.11 and LPIPS of 0.03—roughly 40% lower than the second-best CAAM. These results corroborate that the adaptive NAG momentum and diffusion-based noise scheduling embedded in ours successfully concentrate the perturbation energy on transferable yet perceptually redundant directions, delivering the best known trade-off between transfer strength and visual stealth.

**Fig 4:**
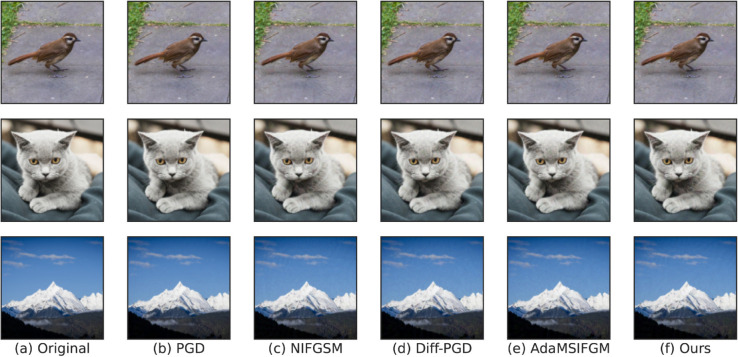
Visualization of adversarial samples. (a) represents the original image and (b)-(f) represent the adversarial examples generated by five methods.

**Qualitative analysis.** We visualize fifteen adversarial examples generated by PGD, NI-FGSM, Diff-PGD, AdaMSI-FGM, and Ours. The original images are shown in Fig 4(a). We choose ResNet34 as the source model. The resulting adversarial examples are displayed in Fig 4(b)–4(f). It is noteworthy that all of these adversarial noises are human-imperceptible. The experiments demonstrate that the proposed method boosts transferability while preserving stealthiness.

#### Parameter sensitivity and computational cost.

The diffusion model used in Diff-AdaNAG is moderately sensitive to the DDIM sampling parameters. Specifically, the number of sampling steps *T*_*s*_ and the steps *K*_*s*_ used in SDEdit should not be too large. In our experiments, using DDIM50 (*T*_*s*_ = 50) with *K*_*s*_ = 1 achieves the best trade-off between attack success rate and imperceptibility. Increasing *T*_*s*_ (e.g., to DDIM100) or enlarging *K*_*s*_ tends to reduce transferability while slightly improving imperceptibility. The parameters adopted in this paper are chosen based on this balance. In addition, we analyze two remaining hyperparameters, $\theta_{0}$ and *η*. The initial hyperparameter $\theta_{0}$ is set to 1, as theoretically it is required to lie within the range (0,1]. The step size *η* is selected through a grid search over [0.001,0.005,0.01,0.05,0.1], and the optimal value of η=0.05 is adopted in our experiments. In terms of computational cost, most of the runtime comes from the diffusion denoising process, while the adaptive Nesterov optimization adds negligible overhead. Although Diff-AdaNAG is somewhat slower than purely gradient-based attacks, it remains computationally feasible and can be efficiently parallelized on GPUs.

## Discussion

The utilization of Diff-AdaNAG for adversarial sample generation can be viewed as analogous to training neural networks via adaptive NAG optimization augmented with a diffusion mechanism. This intrinsic relationship endows the proposed method with transferability that inherits the accelerated convergence properties and stable generalization guarantees native to NAG. The work presented in this paper further inspires the idea that advanced algorithms in optimization can be leveraged to enhance adversarial attack performance. Future convergence proofs for such algorithms will provide theoretical support for the interpretability of adversarial attack algorithms.

Additionally, a key innovation lies in the adaptive stepsize mechanism, which enables automated per-dimension learning rate assignment for adversarial perturbations. This refinement not only optimizes the noise manifold but strategically reduces update magnitudes in perceptually non-salient regions, thereby minimizing $L_{\infty }$-norm distortion while maintaining attack success rates, enabling stealthy adversarial attacks that are imperceptible to the human eye. The introduction of the diffusion mechanism not only significantly enhances the concealment of generated samples but can also be utilized for style-customized adversarial sample generation to adapt to physical-world attacks. This will be pursued as part of our future research.

## Conclusions

This paper introduces an innovative adversarial attack framework that leverages a diffusion-based perturbation synthesis mechanism to generate visually imperceptible noise patterns. By synergistically integrating momentum extrapolation principles with adaptive step-size scaling, the proposed method unlocks the untapped potential of the vanilla Nesterov’s Accelerated Gradient (NAG) optimizer in crafting transferable adversarial examples under black-box constraints. Extensive experiments on the ImageNet dataset validate the effectiveness of our algorithm, demonstrating its ability to combine with various transfer-based black-box adversarial attacks to boost transferability while maintaining the stealthiness of adversarial attacks.

## Supporting information

S1 Table Supplementary Results for Table 2 (a). Attack success rates (%) of adversarial attacks against twelve models. This table contains the supplementary results for the subsection Flexibility.
(XLSX)

S2 Table Supplementary Results for Table 2 (b) and Fig 3. Attack success rates (%) of adversarial attacks against twelve models. This table contains the supplementary results for the subsection Flexibility and Further Comparison with NI-FGSM.
(XLSX)

S3 Table Supplementary Results for Table 2 (c). Attack success rates (%) of adversarial attacks against twelve models. This table contains the supplementary results for the subsection Flexibility.
(XLSX)

S4 Table Supplementary 4esults for Table 3 (a). Attack success rates (%) of adversarial attacks against twelve models. This table contains the supplementary results for the subsection Flexibility.
(XLSX)

S5 Table Supplementary results for Table 3 (b). Attack success rates (%) of adversarial attacks against twelve models. This table contains the supplementary results for the subsection Flexibility.
(XLSX)

S6 Table Supplementary results for Table 3 (c). Attack success rates (%) of adversarial attacks against twelve models. This table contains the supplementary results for the subsection Flexibility.
(XLSX)

S7 Table Supplementary results for Table 4. Attack success rates (%) of adversarial attacks against twelve models. This table contains the supplementary results for the subsection Flexibility.
(XLSX)
